# Assessment of Intra-Individual Variability and Reproducibility in Pancreatic EUS-Guided Elastography

**DOI:** 10.3390/diagnostics15202601

**Published:** 2025-10-15

**Authors:** Bogdan Miutescu, Renata Bende, Felix Bende, Adrian Burdan, Eyad Gadour, Ana Maria Ghiuchici, Mohammed Alomar, Calin Burciu, Mohammed Saad AlQahtani, Roxana Sirli, Alina Popescu, Iulia Ratiu

**Affiliations:** 1 Division of Gastroenterology and Hepatology, Department of Internal Medicine II, “Victor Babes” University of Medicine and Pharmacy, 300041 Timisoara, Romania; miutescu.bogdan@umft.ro (B.M.); bende.felix@umft.ro (F.B.); ghiuchici.anamaria@umft.ro (A.M.G.); sirli.roxana@umft.ro (R.S.); popescu.alina@umft.ro (A.P.); ratiu.iulia@umft.ro (I.R.); 2 Advanced Regional Research Center in Gastroenterology and Hepatology, “Victor Babes” University of Medicine and Pharmacy, 300041 Timisoara, Romania; ghita-adrian.burdan@umft.ro (A.B.); calin.burciu@umft.ro (C.B.); 3 Doctoral School, Faculty of Medicine, “Victor Babes” University of Medicine and Pharmacy, 300041 Timisoara, Romania; 4 Multi-Organ Transplant Centre of Excellence, Liver Transplantation Unit, King Fahad Specialist Hospital, Dammam 32253, Saudi Arabia; eyadgadour@doctors.org.uk (E.G.); al3omar99@gmail.com (M.A.); alqahtanim222@gmail.com (M.S.A.); 5 Department of Medicine, Faculty of Medicine, Zamzam University College, Khartoum 11113, Sudan; 6 Department of Gastroenterology, Faculty of Medicine, Pharmacy and Dental Medicine, “Vasile Goldis” West University of Arad, 310414 Arad, Romania; 7 Department of Surgery, Imam Abdulrahman Bin Faisal University, Dammam 31441, Saudi Arabia

**Keywords:** pancreas, endoscopic ultrasound, body mass index, elastography

## Abstract

**Background**: Shear-wave elastography (SWE) performed during endoscopic ultrasound (EUS) is a promising tool for quantifying pancreatic stiffness, but its intra-session reproducibility remains incompletely defined. **Methods**: In this prospective single-center study, 86 consecutive patients (median age 66 years; 59.3% women) referred for diagnostic EUS underwent EUS-guided point SWE. Ten measurements were acquired from a 10 × 15 mm region of interest in the pancreatic body or tail when the breath was held by a single expert operator. Reproducibility was assessed by comparing the first and last five acquisitions; intra-individual variability was expressed as the coefficient of variation (CV). **Results**: Mean stiffness was 18.5 ± 8.9 kPa (2.31 ± 0.58 m/s). Agreement between early and late measurements was excellent in kPa (ICC = 0.99; r = 0.997; mean bias −0.06 kPa) and moderate in m/s (ICC = 0.61; r = 0.61). The mean CVs were 0.640 for kPa and 0.328 for m/s. Sex, age, and BMI had no significant influence on stiffness or reproducibility. The technical success rate was 97%, with no adverse events. **Conclusions**: EUS-guided point SWE provides highly reproducible pancreatic stiffness measurements within a single session, particularly when expressed in kPa. Demographic factors do not affect stability, supporting its integration into routine EUS practice. Further multicenter studies are needed to establish pathology-specific cut-offs and confirm clinical relevance.

## 1. Introduction

Elastography has emerged as a valuable non-invasive imaging technique for the quantitative assessment of tissue stiffness in various organs, including the liver, breast, thyroid, and, more recently, the pancreas. Shear-wave elastography (SWE), in particular, allows for real-time evaluation of mechanical tissue properties by measuring the propagation speed of shear waves generated by acoustic radiation force. In pancreatic applications, elastography has shown potential in characterizing both focal lesions and diffuse parenchymal changes associated with inflammation, fibrosis, or neoplastic transformation.

The percutaneous elastographic assessment of the pancreas is particularly challenging due to the organ’s retroperitoneal location, small size, deep position, and susceptibility to respiratory motion artifacts, often resulting in suboptimal acoustic windows and limited reproducibility. In contrast, endoscopic ultrasound (EUS) elastography provides more direct and stable access to the pancreas, allowing for higher-resolution imaging and the more consistent acquisition of elastographic data. SWE integrated into EUS platforms enables the direct, intraluminal assessment of pancreatic tissue by measuring the propagation velocity of shear waves induced by acoustic radiation force. This allows the quantification of parenchymal stiffness, which can reflect underlying inflammatory, fibrotic, or neoplastic changes. EUS elastography has shown promise in distinguishing malignant from benign pancreatic lesions and in evaluating diffuse pancreatic diseases, such as chronic pancreatitis or autoimmune pancreatitis [1].

Several recent studies have highlighted the clinical value of EUS elastography as a promising tool for the differential diagnosis of solid pancreatic tumors [2,3,4,5,6]. Qualitative elastographic patterns—such as the presence of predominant blue areas, indicating increased stiffness—have been shown to correlate with malignancy. In the seminal study by Giovanni et al. [2], masses that appeared predominantly blue on elastography were more likely to be malignant, whereas heterogeneous or green-dominant patterns were associated with benign pathology. A larger multicenter study including 121 patients reported a sensitivity of 92.3% and a specificity of 80% for malignancy based on EUS elastographic features [5].

Nonetheless, qualitative elastography remains operator-dependent and may suffer from subjectivity. This limitation is reflected in the discordant results published by Hirche et al. [7], who reported low diagnostic performance with a sensitivity of 41%, specificity of 53%, and overall accuracy of only 45%. To address this, quantitative approaches such as strain ratio and shear-wave measurements were developed, as extensively reviewed elsewhere [1]. In a study by Iglesia-Garcia et al. [8], quantitative elastography achieved superior diagnostic accuracy, with a specificity of 92.9% and overall accuracy of 97.7%, compared to qualitative methods. These findings support the need for standardized and reproducible elastographic protocols, particularly when applied to challenging organs like the pancreas.

Differentiating pancreatic cancer from chronic pancreatitis remains difficult, especially in advanced cases, with EUS accuracy not exceeding 75% and EUS-FNA (Fin-Needle Aspiration) yielding sensitivities between 75 and 92% [9,10,11]. EUS elastography may offer additional value, as it focuses only on the lesion, regardless of surrounding inflammation. While qualitative analysis may misclassify inflammatory masses as malignant in up to 20% of cases—mostly as false positives [4]—quantitative elastography improves diagnostic accuracy and may be useful when EUS-FNA is inconclusive [6].

Despite its growing adoption, data on the reproducibility and intra-individual variability of pancreatic SWE performed during EUS are still limited. Unlike hepatic elastography—where measurement protocols, thresholds, and reproducibility benchmarks are well established—pancreatic elastography lacks standardized acquisition protocols.

In clinical practice, multiple elastographic acquisitions are often recommended to reduce the impact of technical variability and outliers [7,1,8,9,10]. Yet, there is currently no consensus regarding the optimal number of measurements, the best statistical approach to summarize them (mean vs. median), or the acceptable range of intra-individual variation. The intra-observer reproducibility and intra-individual variability of EUS- pSWE pancreatic elastography, especially during the same session and performed by the same operator, are critical for ensuring the method’s diagnostic reliability.

This study aims to evaluate the consistency and variability of pancreatic elastography measurements performed by EUS-guided point shear-wave elastography (pSWE) in a real-life clinical setting. By analyzing repeated measurements obtained during the same examination, we explore the reliability of the method, the potential influence of patient-related factors such as age and sex, and the impact of presenting results in kilopascals (kPa) versus meters per second (m/s). The goal is to better understand how pancreatic elastography behaves in clinical practice and contribute to the standardization and optimization of its use.

## 2. Materials and Methods

### 2.1. Study Design and Ethical Approval

We performed a single-center, prospective observational study in the Gastroenterology Department of the Emergency County Hospital Timisoara, Romania, from 1 January 2024 to 31 March 2025. The protocol conformed to the Declaration of Helsinki and was approved by the institutional ethics committee of “Pius Brînzeu” County Emergency Clinical Hospital (No. 38/11 November 2023). All participants gave written informed consent before enrolment, and the report follows the STROBE recommendations for observational research.

### 2.2. Setting and Study Population

Consecutive adults aged eighteen years or older who were referred for diagnostic EUS of the pancreas during the study period constituted the source population. Patients were included if a complete EUS examination of the pancreas was feasible and at least ten technically valid pSWE measurements could be acquired in a single parenchymal segment during the same session.

The exclusion criteria comprised an inadequate acoustic window or major respiratory artifact, previous pancreatic resection or metallic stents, acute pancreatitis within six weeks, walled-off necrosis or a pseudocyst larger than two centimeters, as large fluid-filled collections are known to preclude valid SWE measurements [11] pregnancy or lactation, uncontrolled coagulopathy defined as an international normalized ratio above 1.5 or a platelet count below 50 × 10^9^ L, and inability to cooperate with breath-hold instructions or to provide informed consent. Eighty-six consecutive patients met all criteria and were enrolled.

### 2.3. Endoscopic Ultrasound Elastography Protocol

All examinations were conducted with an Olympus UCT-180 linear-array echo-endoscope *(*Olympus Medical Systems, Tokyo, Japan*)* linked to a Hitachi Arietta 850 *(Hitachi Medical Systems, Tokyo, Japan)* workstation that incorporates real-time pSWE software (System Version 00-3.2.0). A single investigator performed all measurements, thereby eliminating inter-operator variability. A measurement was considered valid if the reliability index, expressed as Velocity (VsN), was greater than 50%, in line with the manufacturer’s specifications and previous validation studies [12,13,14]. For each patient, ten consecutive EUS-pSWE measurements were obtained from the same pancreatic portion, most frequently at the level of the head and body, depending on the best acoustic window and image stability. The region of interest (ROI) was consistently placed in homogeneous parenchymal areas, avoiding vessels, ducts, and cystic or calcified structures. The procedure followed principles outlined in international guidelines for elastography [15].

### 2.4. Variable Definitions and Data Collection

Shear-wave velocity (c, m s^−1^) represents the speed of transverse wave propagation, whereas Young’s modulus (E, kPa), automatically displayed by the system, is calculated according to the formula E = 3ρc^2^, assuming a soft-tissue density of 1000 kg m^3^ [16]. Demographic data, indication for EUS and comorbidities were recorded prospectively. For each patient, the study database stored the ten raw velocity and elasticity readings, their mean, median, standard deviation and the coefficient of variation, which was defined as the standard deviation divided by the mean of the ten measurements.

### 2.5. Outcomes

The primary outcome was intra-session reproducibility, quantified by comparing the mean of the first five measurements with the mean of the last five measurements in both kPa and m/s, using the intraclass correlation coefficient (ICC). Secondary outcomes included intra-individual variability, reported as the coefficient of variation, agreement between odd- and even-numbered acquisitions, agreement between overall mean and median values, and Bland–Altman bias with 95% limits of agreement.

### 2.6. Statistical Analysis

The distribution of continuous variables was assessed with the Kolmogorov–Smirnov test; normally distributed variables are expressed as mean ± standard deviation, whereas non-normal variables are shown as median with inter-quartile range. Since the elastography values were normally distributed, mean values were used for descriptive statistics and reproducibility analyses, as they best reflect central tendency and allow direct assessment of bias in Bland–Altman plots. Differences between sexes and between patients younger than sixty-five years and those aged sixty-five years or older were examined with independent-samples t-tests or Mann–Whitney U tests, as appropriate. ICCs were calculated with a two-way mixed-effects, single-measurement, absolute-agreement model and interpreted according to literature that designates values above 0.90 as indicating excellent reliability [17]. Pearson’s correlation coefficient quantified linear associations, and Bland–Altman plots assessed bias and limits of agreement. All analyses were performed with MedCalc 19.4 and Microsoft Excel 2019; a two-sided *p*-value below 0.05 defined statistical significance. Missing data were less than two per cent and were handled by pairwise deletion.

## 3. Results

Table 1 compares mean pancreatic stiffness across sex and age strata and shows that demographic factors do not materially influence shear-wave elastography (SWE) read-outs obtained during a single endoscopic ultrasound (EUS) session. Women exhibited slightly higher stiffness than men (20.31 ± 9.49 kPa vs. 17.63 ± 6.62 kPa), but the difference was not significant (*p* = 0.215); analogous non-significance was observed for shear-wave velocity (2.38 ± 0.62 m/s vs. 2.28 ± 0.47 m/s; *p* = 0.481).

Younger participants (< 65 years, *n* = 30) likewise trended toward greater stiffness (20.68 ± 9.13 kPa; 2.43 ± 0.57 m/s) than their older counterparts (≥ 65 years, *n* = 56: 17.03 ± 8.70 kPa; 2.18 ± 0.61 m/s), yet the gap again fell short of statistical significance (*p* = 0.078 for kPa, *p* = 0.067 for m/s).

A total of 86 subjects who underwent EUS for various clinical indications were included in the analysis, comprising 35 men (40.7%) and 51 women (59.3%). The median age was 66 years, with a range from 31 to 89 years. The underlying clinical diagnoses are summarized in Table 2.

**Figure 1:**
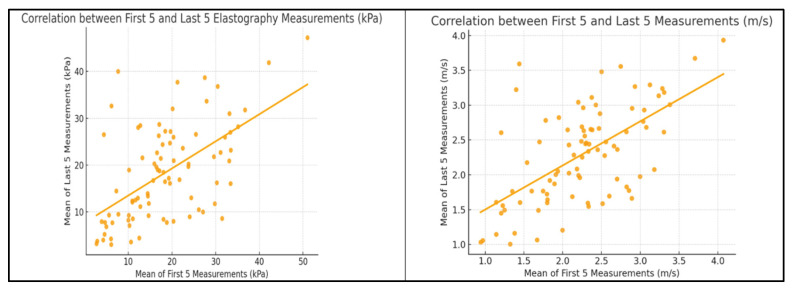
Correlation Between the First Five and Last Five EUS Elastography Measurements Expressed in kPa and m/s.

A strong intra-observer reproducibility was demonstrated when comparing the first five versus the last five elastography measurements expressed in kPa. The ICC was 0.99, and the Pearson correlation coefficient was r = 0.997 (*p* < 0.0001), confirming an excellent agreement between the two series. When comparing the first five versus the last five velocity-based elastography measurements expressed in m/s, the ICC was 0.61 and the Pearson correlation coefficient was r = 0.61 (*p* < 0.0001), indicating a moderate agreement and reproducibility between the two sets (Figure 1).

**Figure 2:**
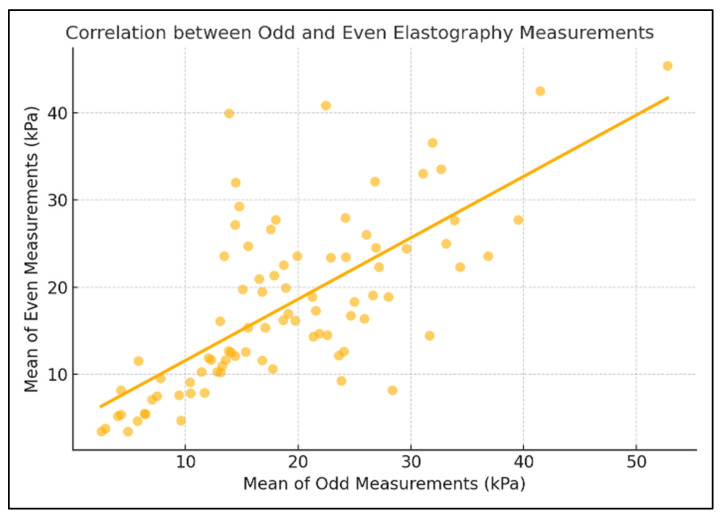
Correlation Between Odd and Even Elastography Measurements.

**Figure 3:**
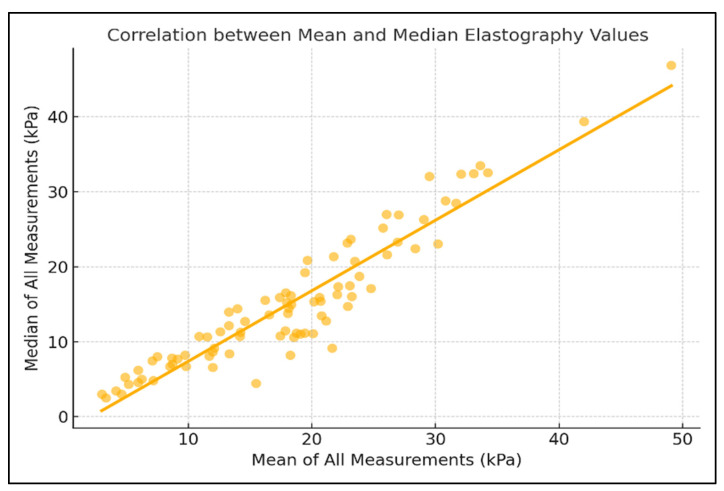
Correlation Between Mean and Median Pancreatic Elastography Measurements (kPa).

To further assess internal consistency, the elastography measurements were divided into odd- and even-numbered sequences. The agreement between these subsets was good, with an ICC of 0.702 and a Pearson correlation coefficient of r = 0.702 (*p* < 0.0001) (Figure 2). In addition, the overall mean and median values across all ten measurements per patient were highly consistent, showing an excellent agreement (ICC = 0.938, r = 0.938, *p* < 0.0001), supporting the robustness of the central tendency measures used in the analysis (Figure 3).

**Figure 4:**
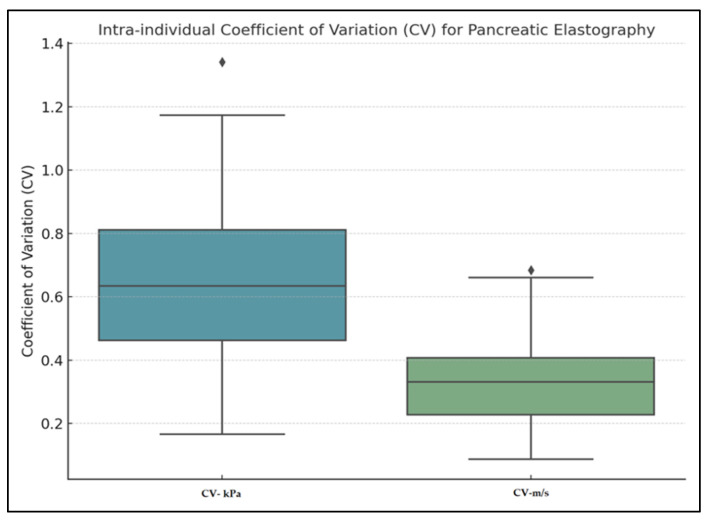
Intra-individual Coefficient of Variation (CV) for pancreatic elastography.

**Figure 5:**
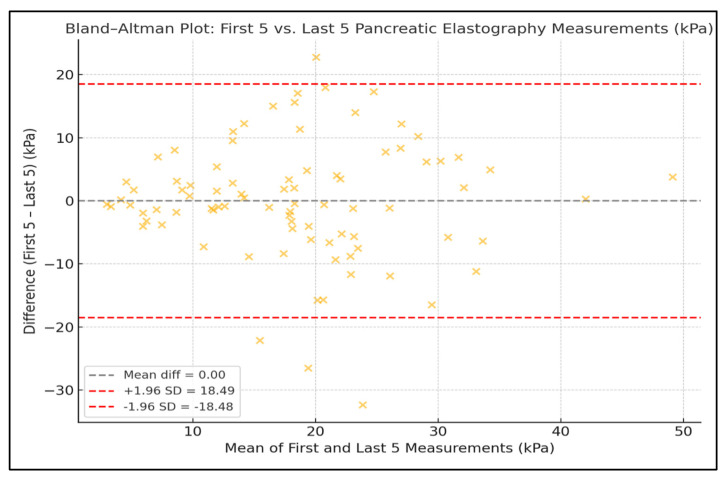
Bland–Altman Plots Showing Agreement Between the First and Last Five Pancreatic Elastography Measurements (kPa).

**Figure 6:**
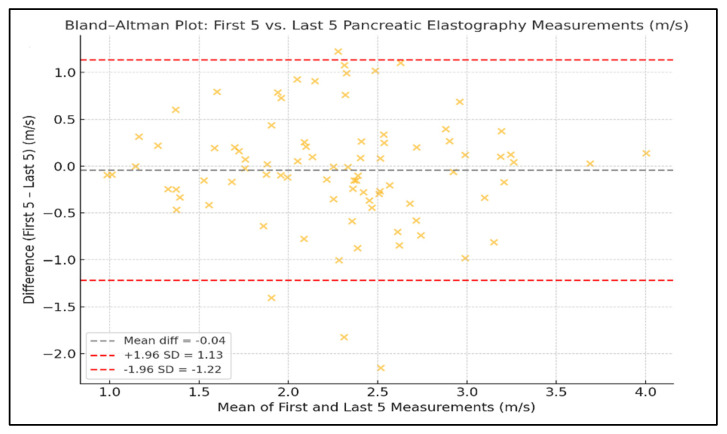
Bland–Altman Plots Showing Agreement Between the First and Last Five Pancreatic Elastography Measurements (m/s).

Intra-individual variability of pancreatic elastography measurements was assessed using the coefficient of variation (CV). The mean CV for values expressed in kilopascals was 0.640 (median 0.634, range: 0.166–1.342), while for values expressed in meters per second, the mean CV was 0.328 (median 0.331, range: 0.087–0.684), as presented in Figure 4. Agreement between the first five and last five pancreatic elastography measurements (in kPa) was further assessed using a Bland–Altman analysis. The mean difference was −0.06 kPa, with limits of agreement ranging from −5.63 to +5.52 kPa. The differences were symmetrically distributed around the mean, indicating a good level of reproducibility across repeated measurements. In the Bland–Altman analysis of the first five versus last five measurements expressed in meters per second, the mean difference was −0.01 m/s, with limits of agreement from −0.43 to +0.41 m/s (Figure 5 and Figure 6).

Table 3 compares intra-session point shear-wave elastography (pSWE) performance across sex and age sub-groups. Mean pancreatic stiffness was numerically higher in women (20.3 kPa) than in men (17.6 kPa) and in participants < 65 years (20.7 kPa) versus those ≥65 years (17.0 kPa), but these differences were small and fell within overlapping standard deviations. Coefficients of variation (≈0.63–0.65 for kPa; ≈0.32–0.34 for m/s) were virtually identical among groups, indicating comparable within-session dispersion. Crucially, reproducibility—quantified by the intraclass correlation coefficient (ICC) between the first and last five measurements—remained excellent for stiffness expressed in kPa (ICC = 0.98–0.99) and moderate but consistent for m/s (ICC ≈ 0.60–0.62) across all comparisons.

Table 4 demonstrates that the body’s habitus has minimal impact on SWE reproducibility. Normal-weight subjects (BMI < 25 kg m^−2^; *n* = 24) showed a mean stiffness of 17.8 ± 8.2 kPa with a coefficient of variation (CV) of 0.62 ± 0.14 and an intraclass correlation coefficient (ICC) of 0.98 between the first and last five measurements. Overweight (*n* = 32) and obese (*n* = 30) groups displayed slightly higher stiffness (18.9 ± 8.9 and 19.7 ± 9.6 kPa) and correspondingly higher CVs (0.64 ± 0.13 and 0.65 ± 0.15), yet ICCs remained excellent at 0.99 for kPa and ≈0.61 for m/s in all strata.

## 4. Discussion

### 4.1. Assessment of Findings and Additional Literature

Our findings confirm that point shear-wave elastography, when performed via EUS, is a feasible and technically reliable method for assessing pancreatic parenchymal stiffness. By overcoming the acoustic limitations of transabdominal imaging, as highlighted in recent comparative reviews [18], the endoscopic approach enables the acquisition of consistent, high-quality elastographic data. In our cohort, pSWE demonstrated excellent intra-observer reproducibility for measurements expressed in kPa, with an intraclass correlation coefficient of 0.99 and a Pearson correlation of 0.997, underscoring the robustness of kPa-based values as a reliable surrogate for tissue stiffness.

Pancreatic elastography holds significant clinical potential in the evaluation of both diffuse parenchymal changes—such as those seen in chronic pancreatitis—and focal pancreatic masses, facilitating the non-invasive differentiation between benign and malignant lesions [19,20]. However, before these diagnostic distinctions can be meaningfully applied in clinical practice, it is essential to understand how reproducible, consistent, and technically reliable the method is. While the transabdominal approach is often limited by suboptimal acoustic windows and anatomical constraints, many of these challenges can be overcome through endoscopic ultrasound guidance, which provides more direct and stable access to the pancreas and allows for high-resolution, reproducible elastographic measurements [21].

These findings are consistent with previously published data supporting the reliability of elastography in the evaluation of chronic pancreatitis (CP). A retrospective study using semi-quantitative elastography in 96 patients demonstrated significantly different elastographic values across Rosemont classification stages (*p* < 0.001), indicating the method’s capacity to reflect progressive parenchymal changes [22]. Similarly, another investigation in 84 patients showed a significant correlation between pancreatic stiffness and the number of EUS features (rs = 0.47), with an AUROC of 0.77 for distinguishing consistent/suggestive CP from normal/indeterminate findings [23]. Additionally, shear-wave measurements performed via EUS have shown promising correlation with CP staging criteria, such as those of the Japan Pancreas Society. In a cohort of 40 patients, higher stiffness values were associated with more advanced disease stages, and AUROC values of up to 0.92 were reported for CP diagnosis [24]. Although these studies did not primarily assess intra-observer reproducibility, the strong correlations and high diagnostic accuracy suggest that elastographic measurements are stable and clinically informative when properly applied.

In contrast, measurements expressed in m/s showed only moderate reproducibility (ICC and Pearson r = 0.61). Although m/s reflects the direct velocity of shear-wave propagation, it is less sensitive to subtle tissue stiffness changes, especially at higher values. The non-linear conversion to kPa, based on Young’s modulus (E = 3ρc^2^), amplifies these differences, enhancing the dynamic range and improving differentiation between normal and pathological tissue. This fundamental advantage of expressing stiffness in kPa has been discussed in recent reviews on elastography biophysics [1].

Paradoxically, while kPa values show higher variability (mean CV = 0.640 vs. 0.328 for m/s), they offer better diagnostic sensitivity and clinical interpretability—particularly in the pancreas, where shear-wave velocities tend to cluster in narrow ranges. Small changes in m/s can result in disproportionately larger shifts in kPa, making the latter more useful despite its greater variability.

This preference for kPa-based measurements is further supported by evidence showing that kPa values provide higher specificity and better performance in quantifying tissue heterogeneity, particularly when evaluating standard deviation and whole-lesion variability, even when overall diagnostic accuracy appears similar between kPa and m/s [25]. In addition, the better reproducibility observed for kPa may also reflect differences in numerical scaling and rounding precision between the two units, with kPa values providing a smoother distribution and less sensitivity to minor computational fluctuations.

Sub-analysis comparing odd- and even-numbered measurements showed good internal consistency (ICC and r = 0.702), and the agreement between mean and median values across the 10 acquisitions was excellent (ICC = 0.938, r = 0.938), indicating that either central tendency measure may be used reliably in clinical reporting.

The Bland–Altman analysis further supported the stability of repeated measurements, showing narrow limits of agreement (±5.6 kPa and ±0.43 m/s) and no systematic bias between early and late measurement sets. These findings reinforce the method’s intra-session reproducibility and its applicability in routine clinical practice.

With respect to demographic variables, no statistically significant differences in elastography values were observed between male and female patients or between age groups (<65 vs. ≥65 years). However, there was a non-significant trend toward higher stiffness values in younger individuals, which may suggest age-related structural pancreatic changes that warrant further investigation.

EUS-SWE is an emerging technique with demonstrated feasibility and safety in the evaluation of pancreatic pathology [26,27,28]. A retrospective study involving 50 patients reported that measurements were most reliable in the pancreatic body and showed the highest diagnostic accuracy for CP (AUC 0.87), with region-specific vs. cut-offs between 2.10 and 2.33 m/s. Increasing evidence supports the clinical utility of EUS-SWE in the diagnosis and severity assessment of CP [29], autoimmune pancreatitis [30], and fatty pancreas [31,32].

Our intra-session intraclass correlation coefficient (ICC) of 0.99 for stiffness expressed in kPanot only satisfies the “excellent” reliability threshold but also aligns closely with values reported in large transabdominal series. Huang et al. obtained an intra-operator ICC of 0.96 in 387 healthy volunteers studied with 2-D SWE and likewise found that inter-operator agreement fell to “moderate–good” levels—mirroring the drop we observed when switching from kPa to meters per second (m/s). Zhuo et al., using a similar 2-D SWE platform, confirmed excellent same-day repeatability (ICC 0.93) for the pancreatic head and body, although tail measurements were less robust. Taken together, these external data reinforce our observation that the endoscopic approach, which circumvents respiratory artifact and offers shorter acoustic paths, delivers reproducibility at least as good as that seen with carefully executed transabdominal protocols [14,33,34,35].

The absolute stiffness values recorded here provide a clinical context for threshold-setting efforts. Our mean pancreatic stiffness of 18.5 ± 8.9 kPa (≈2.31 m/s) is substantially higher than the 6.46 ± 2.87 kPa reported by Huang et al. in healthy adults, underscoring the influence of underlying pathology in an unselected EUS population. Disease-specific elevations are equally evident in other cohorts: Almutairi demonstrated a 50% rise in mean velocity among patients with type 1 diabetes mellitus versus controls, while Hristov delineated a gradient from chronic pancreatitis (1.75 m/s) to pancreatic ductal adenocarcinoma (2.93 m/s). Placing our mixed-indication average midway between these extremes highlights both the need for pathology-stratified cut-offs and the potential value of standardized, depth-controlled acquisition protocols to harmonize results across centers [14,29,32].

### 4.2. Study Limitations

This study has several limitations that merit consideration. Its primary objective was to assess the technical aspects of EUS-pSWE, focusing on intra-individual variability and reproducibility during a single session. Consequently, we did not analyze elastography values in relation to specific pancreatic pathologies or correlate them with histological findings or clinical outcomes. The diagnostic utility of the method—although promising—remains to be validated in pathology-stratified cohorts, and no cut-off values for clinical decision-making were proposed in the present work. In this regard, we acknowledge that the absence of stratified analyses by disease etiology represents a limitation of the current study and will be specifically addressed in future research.

Furthermore, although the cohort included consecutive patients referred for EUS, the sample size was relatively limited, and the population was heterogeneous in terms of clinical indications and suspected pathology. While this reflects real-life clinical practice, it may reduce the ability to extract disease-specific insights or generalize the findings across all patient groups. Additionally, the entire examination protocol was performed by a single experienced operator in a single center, which enhances internal consistency but limits extrapolation to other settings where inter-operator variability may be significant. Another limitation of the study is the lack of external interobserver reproducibility assessment, as all measurements were performed by a single experienced operator; this aspect will be explored in future multicenter evaluations.

Future multicenter studies, ideally conducted according to emerging standardized EUS-SWE acquisition protocols, will be essential to ensure methodological comparability and to define reliable diagnostic thresholds for clinical application.

## 5. Conclusions

These findings demonstrate that EUS-pSWE is a reproducible, technically reliable, and clinically promising method for quantifying pancreatic stiffness, particularly when applied under controlled and standardized conditions. The high level of intra-observer agreement—especially for stiffness expressed in kPa—underscores its potential role in routine EUS protocols. However, further prospective, multicenter studies involving larger and pathology-specific cohorts are necessary to define diagnostic thresholds, evaluate performance metrics, and establish the role of EUS-pSWE within structured diagnostic algorithms.

## Figures and Tables

**Table 1:** Comparison of Mean Pancreatic Elastography Values by Gender and Age Group.

Variables	Group 1	Group 2	*p*-Value
Gender comparison			
Measurements unit	Females	Males	
kPa	20.31 ± 9.49	17.63 ± 6.62	0.215
m/s	2.38 ± 0.62	2.28 ± 0.47	0.481
**Age group comparison**			
Measurements unit	<65 years (*n* = 30)	**≥65 years (** ***n*** ** = 56)**	
kPa	20.68 ± 9.13	17.03 ± 8.70	0.078
m/s	2.43 ± 0.57	2.18 ± 0.61	0.067

kPa—kilopascals; m/s—meters per second.

**Table 2:** Distribution of Diagnoses in the Study Cohort (*n* = 86).

Diagnostic	*n*	%
Pancreatic neoplasm	30	34.9
Suspected choledocholithiasis (not confirmed)	30	34.9
Choledocholithiasis	12	14.0
Acute pancreatitis	4	4.7
Chronic pancreatitis	3	3.5
Pancreatic cyst	3	3.5
Neuroendocrine tumor	2	2.3
Malignant ampullary tumor	1	1.2
Benign ampullary tumor	1	1.2
Total	86	100

**Table 3:** Sub-group analysis of intra-session variability and reproducibility of pancreatic EUS-pSWE measurements according to sex and age.

Sub-Group	*n*	Mean Stiffness ± SD (kPa)	CV (kPa) ± SD	ICC (First 5 vs. Last 5, kPa)	Mean Velocity ± SD (m/s)	CV (m/s) ± SD	ICC (First 5 vs. Last 5, m/s)
Sex							
Females	51	20.3 ± 9.5	0.64 ± 0.14	0.99	2.38 ± 0.62	0.33 ± 0.10	0.61
Males	35	17.6 ± 6.6	0.63 ± 0.13	0.98	2.28 ± 0.47	0.32 ± 0.09	0.60
Age (years)							
< 65	30	20.7 ± 9.1	0.65 ± 0.15	0.99	2.43 ± 0.57	0.34 ± 0.11	0.62
≥ 65	56	17.0 ± 8.7	0.63 ± 0.14	0.98	2.18 ± 0.61	0.32 ± 0.10	0.60

CV = coefficient of variation (SD ÷ mean of ten sequential acquisitions); ICC = intraclass correlation coefficient (two-way mixed-effects model, absolute agreement). All sub-groups demonstrated excellent reproducibility for stiffness expressed in kilopascals (ICC ≥ 0.98) and moderate reproducibility for measurements expressed in meters per second (ICC ≈ 0.60). Slight numerical differences in variability (CV) were not statistically significant, indicating that neither sex nor age materially affects intra-session stability of pancreatic EUS-pSWE readings.

**Table 4:** Influence of body-mass-index (BMI) category on intra-individual variability and reproducibility of pancreatic EUS-pSWE measurements.

BMI Category (kg m^−2^)	*n* (Patients)	Mean Stiffness kPa ± SD	Mean CV (kPa) ± SD	ICC (First 5 vs. Last 5, kPa)	Mean Velocity m s^−1^ ± SD	Mean CV (m/s) ± SD	ICC (First 5 vs. Last 5, m/s)
Normal (<25)	24	17.8 ± 8.2	0.62 ± 0.14	0.98	2.25 ± 0.48	0.31 ± 0.09	0.6
Overweight (25–29.9)	32	18.9 ± 8.9	0.64 ± 0.13	0.99	2.30 ± 0.54	0.33 ± 0.10	0.62
Obese (≥30)	30	19.7 ± 9.6	0.65 ± 0.15	0.99	2.34 ± 0.61	0.34 ± 0.11	0.61

SD = standard deviation; CV = coefficient of variation; ICC = intraclass correlation coefficient. Higher BMI was associated with slightly longer shear-wave velocity and kPa values, but variability (CV) and reproducibility (ICC) remained stable across BMI strata, indicating that increased submucosal adiposity did not meaningfully degrade EUS-pSWE performance in this cohort.

## Data Availability

The data are available upon request.
